# Development and validation of a nomogram for predicting peripheral atherosclerosis in early-stage type 2 diabetic kidney disease: a retrospective hospital-based study

**DOI:** 10.3389/fendo.2026.1870637

**Published:** 2026-07-07

**Authors:** Sixu Xin, Xiaomei Zhang, Jianbin Sun

**Affiliations:** Department of Endocrinology, Peking University International Hospital, Beijing, China

**Keywords:** Chinese visceral adiposity index, diabetic kidney disease, nomogram prediction model, peripheral atherosclerosis, type 2 diabetes mellitus

## Abstract

**Introduction:**

Microvascular disease is a common complication of type 2 diabetes mellitus (T2DM). As a typical microvascular complication, diabetic kidney disease (DKD) interacts with peripheral atherosclerosis (AS) and significantly increases the risk of adverse cardiovascular events.

**Objectives:**

To investigate the risk factors for peripheral AS in early-stage T2DM-DKD patients, and to construct an individualized nomogram prediction model.

**Methods:**

A single-center, retrospective study was conducted involving 392 T2DM patients with early-stage DKD admitted to Peking University International Hospital from March 2015 to August 2021. According to the results of peripheral arterial ultrasound examination, they were divided into peripheral AS group (AS+DKD group) (n = 294) and DKD-only group (n = 98). Clinical data and laboratory parameters were collected, and CVAI was calculated. Univariate and multivariate logistic regression analyses were used to identify independent factors associated with peripheral AS. A nomogram model was constructed using R software, and the predictive performance of the model was evaluated by receiver operating characteristic (ROC) curves, calibration curves, and decision curve analysis (DCA).

**Results:**

The incidence of peripheral AS in early-stage T2DM-DKD patients was 75.00%. Univariate analysis showed that the AS+DKD group had higher Age, longer duration of diabetes, higher CVAI, HDL-C and sCr level, as well as lower BMI, DBP, fasting C-peptide (FCP), 2-hour C-Peptide, TC, TG, LDL-C, ALT, AST, and eGFR levels (all P < 0.05). Multivariate logistic regression analysis revealed that elevated CVAI (OR = 1.034, 95% CI: 1.010- 1.058), increased Age (OR = 1.054, 95% CI: 1.018- 1.090), and decreased FCP (OR = 0.771, 95% CI: 0.608- 0.978) were independent risk factors for peripheral AS in early-stage T2DM-DKD patients (all P < 0.05). The AUC values for the three models, included Age only, FCP+ Age, and CVAI+ FCP+ Age, were 0.814 (95% CI: 0.765-0.863), 0.825 (95% CI: 0.776-0.874), and 0.837 (95% CI: 0.790-0.884), with a sensitivity of 87.4%, 85.0%, 87.1% and a specificity of 62.2%, 68.4%, 68.4%, respectively. Pairwise DeLong tests revealed that the AUC of the combined model (Age + FCP + CVAI, AUC = 0.837) was significantly larger than those of both the Age + FCP model (AUC difference = 0.0120, 95% CI: 0.000- 0.024, p = 0.0431) and Age alone (AUC difference = 0.0229, 95% CI: 0.003- 0.043, p = 0.0274). No significant difference was observed between Age alone and the Age + FCP model (p = 0.2295). A nomogram model was constructed based on the above three indicators to visualize the risk of AS in early-stage T2DM-DKD patients. The bootstrap-corrected calibration curve showed acceptable agreement between predicted probabilities and observed frequencies. The DCA curve confirmed significant clinical net benefit of the model.

**Conclusions:**

CVAI alone shows limited predictive value for peripheral AS in early-stage T2DM-DKD patients. However, a combined nomogram incorporating CVAI, Age, and FCP demonstrates good discrimination, calibration, and clinical utility, facilitating early identification of high-risk patients and improving prognosis.

## 1 Introduction

Diabetes is a chronic metabolic disease that poses a threat to public health (1, 2). Diabetic kidney disease (DKD) is one of the most common and severe chronic complications in patients with diabetes mellitus (DM). According to a 2021 report by the International Diabetes Federation, the number of adults with DM worldwide is projected to increase from 537 million to 783 million by 2045, and 20%-40% of individuals with DM will develop DKD (3). Data from the 2016 Annual Report of the China Kidney Disease Network indicate that DKD is the most common cause of chronic kidney disease (CKD) among hospitalized patients in China, accounting for 26.7% of cases (4). The detrimental impact of DKD cannot be overlooked, as it not only leads to chronic renal insufficiency and end-stage kidney disease (ESKD), necessitating dialysis or kidney transplantation (5), but also increases the risk of cardiovascular and cerebrovascular events, severely impairs patients’ quality of life, and elevates mortality rates. Metabolic disturbances in blood glucose, lipids, and blood pressure in DKD patients promote the initiation and progression of atherosclerosis (AS). Conversely, AS can induce renal vascular lesions, exacerbate renal ischemia and hypoxia, and further impair kidney function. A close interaction exists between these processes, in which metabolic disorders, kidney injury, and vascular pathology mutually reinforce each other, forming a vicious cycle that significantly increases the risk of cardiovascular events and death. Recent studies have shown that, compared with patients without DKD, biopsy-proven individuals with type 1 or type 2 DM who develop DKD have significantly higher rates of cardiovascular event and mortality (6), severely affecting the prognosis of DKD patients and imposing a substantial disease burden (7). Therefore, early identification of risk factors for AS in DKD patients and the construction of a predictive warning model are of great clinical importance.

Abnormal accumulation of visceral fat is a core pathological link connecting glucolipid metabolic disorders, kidney injury, and vascular lesions. Visceral adipose tissue not only directly increases the risk of cardiovascular disease (CVD) but also exerts its effects through pathways such as promoting inflammation, oxidative stress, and aberrant secretion of adipocytokines (8, 9). However, traditional obesity indicators inadequately reflect the body fat distribution characteristics of Asian populations. And imaging-based assessments have limitations including radiation exposure, high cost, and long processing time (10). Consequently, non-invasive and cost-effective surrogate markers such as the Chinese Visceral Adiposity Index (CVAI) have been developed. The CVAI is designed to reflect the unique body fat distribution of Asian populations and has been validated as an effective tool for evaluating visceral obesity and predicting the risk of DM and CVD (11–13).

This single-center retrospective study aims to analyze the association between CVAI and peripheral AS in early-stage T2DM-DKD patients and to develop an individualized risk prediction model, thereby providing evidence-based support for cardiovascular risk stratification and clinical decision making in early-stage T2DM-DKD patients, and improving their prognosis.

## 2 Materials and methods

### 2.1 Ethics statement

The study was approved by the Ethics Committee of the Peking University International Hospital and was conducted in accordance with the ethics standards of institutional and national research committees and the 1964 Helsinki Declaration and its later amendments or comparable ethics standards. The study was a retrospective analysis; therefore, the requirement for written informed consent was waived.

### 2.2 Research subjects

This single-center, retrospective study included 392 patients diagnosed with early-stage T2DM-DKD who were hospitalized at Peking University International Hospital between March 2015 and August 2021.

Inclusion criteria were as follows: (1) Age ≥ 18 years; (2) patients met the diagnostic criteria for DKD; (3) patients were classified as having early-stage DKD, defined as persistent microalbuminuria with 30mg/g ≤ a urinary albumin-to-creatinine ratio (UACR) <300 mg/g; (4) all patients underwent carotid and/or lower extremity arterial ultrasound examinations upon admission; (5) complete data for all key variables (including Age, BMI, waist circumference, TG, HDL-c, FCP, and outcome variables) were included.

Exclusion criteria were: (1) patients with macroalbuminuria (UACR ≥ 300 mg/g); (2) patients receiving peritoneal or hemodialysis; (3) patients with type 1 diabetes (T1D) or other types of DM, such as gestational DM; (4) presence of acute diabetic complications, such as diabetic ketoacidosis or hyperosmolar hyperglycemic state; (5) known primary renal parenchymal diseases (e.g., glomerulonephritis, interstitial nephritis); (6) recent urinary tract infection or current use of medications known to affect renal function (e.g., nonsteroidal anti-inflammatory drugs, aminoglycoside antibiotics); (7) concomitant acute cardiovascular or cerebrovascular events, acute infection, severe anemia or massive blood loss, malignancy, autoimmune diseases, severe hepatic or renal insufficiency, hypothyroidism, Cushing’s syndrome, etc.; (8) pregnancy or lactation;

### 2.3 Methods

#### 2.3.1 Clinical conditions

General clinical data were collected for all study participants: sex, age, smoking history, alcohol consumption history, height, weight, body mass index (BMI), waist circumference (WC), hip circumference (HC), duration of DM, past medical history, systolic blood pressure (SBP), diastolic blood pressure (DBP) at admission, and current medication status, including glucose-lowering agents, antihypertensive drugs, and statins.

#### 2.3.2 Laboratory biochemical indices

Laboratory biochemical indices included fasting plasma glucose (FPG), 2-hour postprandial plasma glucose (2hPPG), glycosylated hemoglobin (HbA1c), total cholesterol (TC), triglycerides (TG), low-density lipoprotein cholesterol (LDL-C), high-density lipoprotein cholesterol (HDL-C), serum creatinine (sCr), estimated glomerular filtration rate (eGFR), alanine aminotransferase (ALT), aspartate aminotransferase (AST), fasting insulin (FINS), 2-hour postprandial insulin (2hINS), fasting C-Peptide (FCP), and 2-hour C-Peptide (2hCP).

FCP and 2hCP were included in the analysis based on the following considerations: (1) Reflection of endogenous insulin secretion function: In patients with T2DM, particularly those receiving exogenous insulin therapy, C-peptide more accurately reflects endogenous insulin secretion than insulin itself, as it is not subject to interference from exogenous insulin; (2) Pathophysiological association with insulin resistance (IR) and DKD: Previous studies (14) have demonstrated that FCP levels correlate with the degree of IR, and elevated C-peptide levels may participate in the pathogenesis and progression of DKD through mechanisms such as promoting inflammatory responses and mesangial cell proliferation. Therefore, FCP was incorporated into the analysis as a complementary indicator reflecting both pancreatic β-cell function and IR status.

#### 2.3.3 Formula calculations

BMI = Weight (kg)/Height² (m²).

Waist-to-hip ratio (WHR)= WC (cm)/HC (cm).

CVAI (male)= -267.93 + 0.68 × age + 0.03 × BMI + 4.00 × WC + 22.00 × log10(TG) − 16.32 × HDL-C.

CVAI (female)= -187.32 + 1.71 × age + 4.23 × BMI + 1.12 × WC + 39.76 × log10(TG) − 11.66 × HDL-C.

#### 2.3.4 Diagnostic criteria for DKD

DKD is characterized by persistently elevated urinary albumin excretion and/or a progressive decline in kidney function. According to Clinical guideline for the prevention and treatment of DKD in China (2021 edition) and internationally recognized standards (15), the diagnosis is established by the presence of either a UACR ≥ 30 mg/g or an eGFR < 60 mL/min/1.73 m², persisting for more than 3 months, after the exclusion of other primary causes of kidney disease.

#### 2.3.5 Diagnostic criteria for peripheral AS and grouping method

Carotid and lower extremity arterial ultrasonography was performed on all patients using a Phillips iE33 (Washington, DC, USA) color Doppler ultrasound diagnostic system with a probe frequency of 11 MHz. The examinations were conducted by senior ultrasound physicians from our hospital. The presence of vascular sclerosis, plaque, or sclerotic occlusion on ultrasound findings was defined as atherosclerotic plaque formation. Patients with these findings were assigned to AS+DKD group, while those without atherosclerotic plaque formation were assigned to the DKD-only group.

### 2.4 Statistical analysis

Data were analyzed using SPSS software (version 31.0) and R software (version 4.2.1). For all analyses, the percentages of missing values were lower than 5%. We used multiple imputation based on five replications for individuals with missing data about covariates. Continuous variables were expressed as mean ± standard deviation (x ± s) and compared using the t-test. Categorical variables were expressed as n (%) and compared using the chi-square test. Before performing multivariate logistic regression analysis, the variance inflation factor (VIF) was used to assess multicollinearity among candidate variables. A VIF > 10 or tolerance < 0.1 indicated the presence of severe multicollinearity, necessitating variable combination or exclusion. Binary multivariate logistic regression was employed to identify influencing factors for peripheral AS, and the receiver operating characteristic (ROC) curve was plotted. The discriminative ability of the models was evaluated by calculating the area under the curve (AUC), sensitivity, and specificity. A nomogram model was constructed using the rms package in R software. Internal validation was performed using K-fold cross-validation, and the calibration curve and concordance index (C-index) were calculated. Additionally, decision curve analysis (DCA) was performed to evaluate the clinical utility and net benefit threshold range of the model. A P-value < 0.05 was considered statistically significant.

## 3 Results

### 3.1 Baseline characteristics

A total of 392 patients meeting the above inclusion and exclusion criteria were enrolled, including 245 males and 147 females, with a mean age of 57.01 ± 15.47 years. The mean duration of DM was 10.25 ± 8.23 years. A history of smoking was present in 45.66% of patients, and a history of alcohol consumption in 26.02%. The proportion of patients with concomitant hypertension was 62.76%, and with concomitant hyperlipidemia was 43.88%. Of the 392 patients with early T2DM-DKD, 85.71% were on glucose-lowering therapy, 32.91% on antihypertensive therapy, and 28.57% on statin lipid-lowering agents (**Table 1**).

**Table 1:** Baseline characteristics of the study participants with and without peripheral AS.

Variable	DKD-only (n=98)	AS+DKD (n=294)	t (X2)	p
Sex			0.294	0.588
Male	59(60.20%)	186 (63.27%)		
Female	39(39.80%)	108 (36.73%)		
Age (year)	43.66 ± 13.79	61.46 ± 13.30	-11.366	0.000
Duration (year)	5.61 ± 6.65	11.80 ± 8.13	-6.807	0.000
WC (cm)	97.96 ± 13.0	96.43 ± 10.38	1.187	0.118
WHR	0.95 ± 0.07	0.95 ± 0.06	0.152	0.439
BMI (kg/m2)	27.51 ± 4.95	26.08 ± 3.71	3.030	0.001
Smoking history	42(42.86%)	137(46.60%)	0.415	0.520
Alcohol consumption history	22(22.45%)	80(27.21%)	0.866	0.352
Glucose-lowering therapy	91(92.86%)	245(83.33%)	5.444	0.02
Antihypertensive therapy	29(29.59%)	199(67.69%)	43.835	0.000
Statin use	9(9.18%)	103(35.03%)	24.067	0.000
SBP (mmHg)	134.81 ± 19.85	137.36 ± 17.87	-1.190	0.117
DBP (mmHg)	82.89 ± 12.66	79.51 ± 11.10	2.513	0.006
CVAI	140.41 ± 50.52	149.30 ± 39.79	-1.785	0.037
HbA1C (%)	9.34 ± 1.85	9.16 ± 1.89	0.797	0.213
FPG (mmol/L)	10.04 ± 3.48	9.69 ± 3.65	0.830	0.204
2hPPG (mmol/L)	13.39 ± 4.55	13.46 ± 4.16	-0.134	0.447
FINS	16.94 ± 17.58	18.28 ± 45.51	-0.285	0.388
2hINS	51.53 ± 54.47	49.92 ± 72.76	0.201	0.420
FCP	3.15 ± 1.71	2.38 ± 1.33	4.610	0.000
2hCP	6.22 ± 4.23	5.26 ± 3.37	2.280	0.012
TC (mmol/L)	4.64 ± 1.42	4.34 ± 1.27	1.950	0.026
TG (mmol/L)	2.96 ± 2.76	2.18 ± 1.74	3.260	0.001
HDL (mmol/L)	0.91 ± 0.22	0.97 ± 0.23	-2.325	0.010
LDL (mmol/L)	2.70 ± 1.11	2.53 ± 0.97	1.440	0.075
ALT (U/L)	37.07 ± 28.78	22.48 ± 18.69	5.779	0.000
AST (U/L)	29.87 ± 26.98	20.87 ± 10.75	4.714	0.000
sCr (umol/L)	66.66 ± 22.79	71.97 ± 23.3	-1.959	0.025
eGFR	107.20 ± 23.84	91.23 ± 21.54	6.189	0.000

### 3.2 Univariate analysis of early-stage T2DM-DKD with peripheral AS

Peripheral arterial ultrasound results of the 392 T2DM patients with early-stage DKD showed that 294 patients (75%) had atherosclerotic plaque formation. These 294 patients were assigned to the peripheral AS group (AS+DKD group), while the remaining 98 patients without peripheral atherosclerotic plaque formation were assigned to the DKD-only group. The results showed that patients in the AS+DKD group were older and had a longer duration of DM, and higher CVAI, HDL, and sCr levels (P < 0.05). Conversely, they had lower BMI, DBP, FCP, 2hCP, TC, TG, LDL-C, ALT, AST, and eGFR levels (P < 0.05) (**Table 1**).

### 3.3 Multivariate logistic regression analysis of peripheral AS in early-stage T2DM-DKD patients

Multicollinearity diagnostics demonstrated that the VIF for all continuous variables was less than 5 (range: 1.191–3.133), indicating the absence of severe multicollinearity. Using the presence or absence of peripheral AS in patients as the dependent variable, variables with P < 0.05 in the univariate analysis were entered into the multivariate logistic regression model. The results showed that increased CVAI (OR = 1.034, 95% CI: 1.010, 1.058) and Age (OR = 1.054, 95% CI: 1.018, 1.090), together with decreased FCP (OR = 0.771, 95% CI: 0.608, 0.978), were risk factors for peripheral AS in early-stage T2DM-DKD (P < 0.05) (**Table 2**).

**Table 2:** Multivariate regression analysis of the associations between CVAI and peripheral AS.

Variable	B	S.E.	P	OR	95%CI
CVAI	0.033	0.012	0.005	1.034	(1.010,1.058)
Age	0.052	0.017	0.003	1.054	(1.018, 1.090)
Duration	0.019	0.026	0.453	1.020	(0.969, 1.073)
WC	-0.076	0.044	0.085	0.927	(0.850, 1.011)
BMI	-0.083	0.069	0.233	0.921	(0.804, 1.055)
Glucose-lowering therapy	-0.854	0.492	0.082	0.426	(0.162, 1.116)
Antihypertensive therapy	0.659	0.596	0.269	1.933	(0.601, 6.214)
Statin use	0.380	0.490	0.438	1.462	(0.559, 3.821)
DBP	0.008	0.015	0.567	1.008	(0.980, 1.038)
FCP	-0.260	0.121	0.032	0.771	(0.608, 0.978)
TG	-0.110	0.076	0.149	0.896	(0.771, 1.040)
HDL	0.965	0.825	0.242	2.624	(0.521,13.218)
ALT	-0.011	0.006	0.084	0.989	(0.976, 1.002)
eGFR	0.016	0.010	0.103	1.016	(0.997, 1.036)

### 3.4 Development and performance evaluation of a nomogram prediction model for peripheral AS in early-stage T2DM-DKD patients

**Figure 1:**
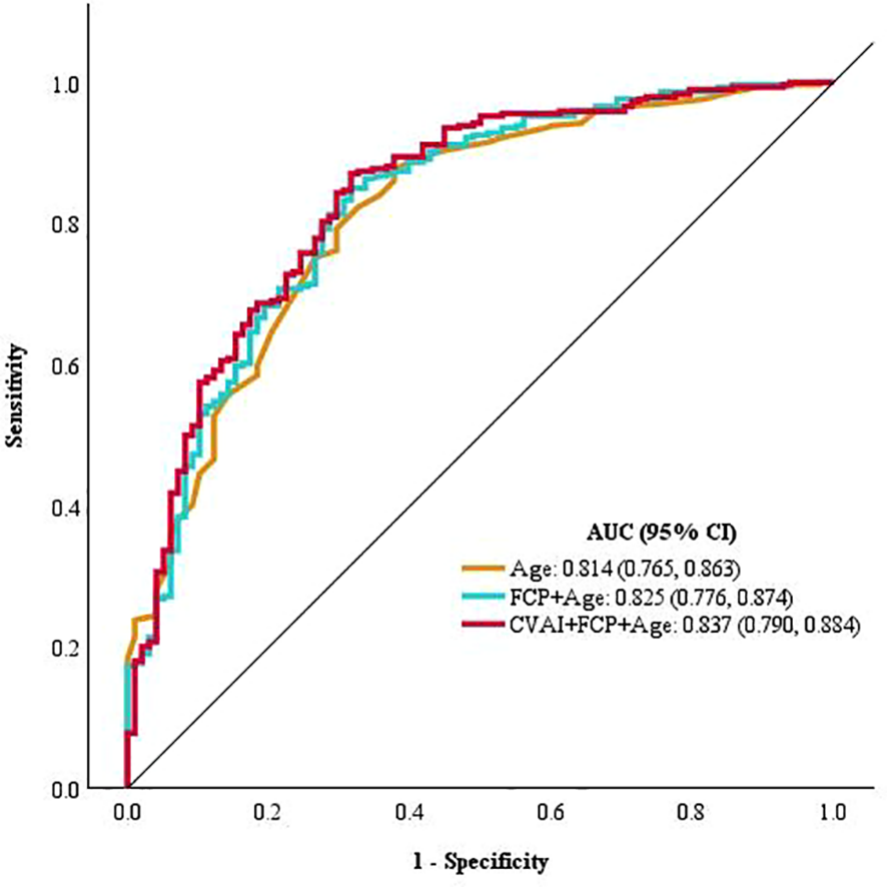
ROC curves for age alone, FCP combined with age, and the nomogram model (CVAI + FCP + Age) in diagnosing peripheral AS in early-stage T2DM-DKD patients. AUC values (95% CI) were 0.814 (0.765- 0.863) for Age alone, 0.825 (0.776- 0.874) for FCP + Age, and 0.837 (0.790- 0.884) for the nomogram model, indicating optimal discriminative performance of the combined model.

**Figure 2:**
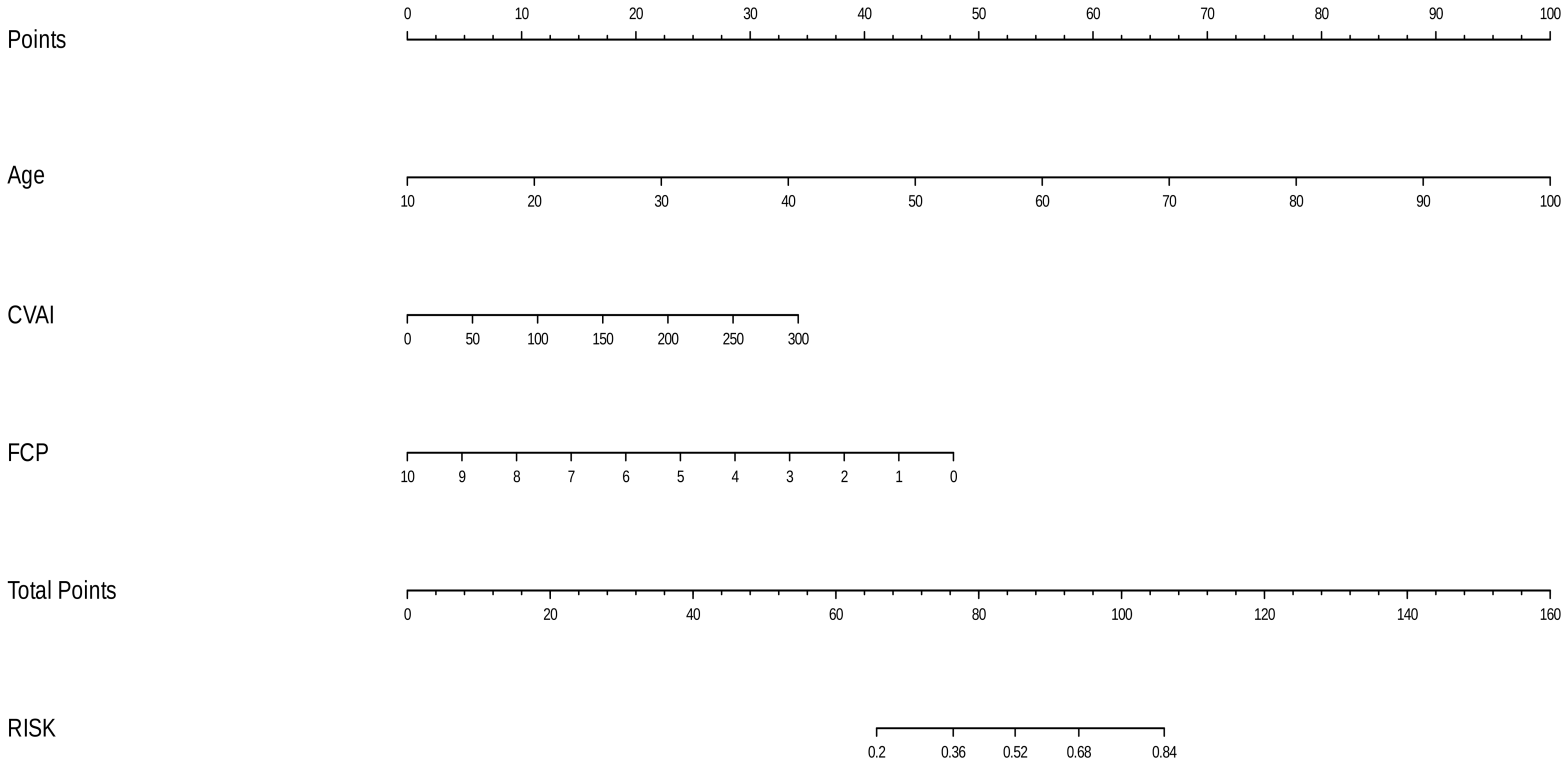
Nomogram model for peripheral AS in early-stage T2DM-DKD patients.

**Figure 3:**
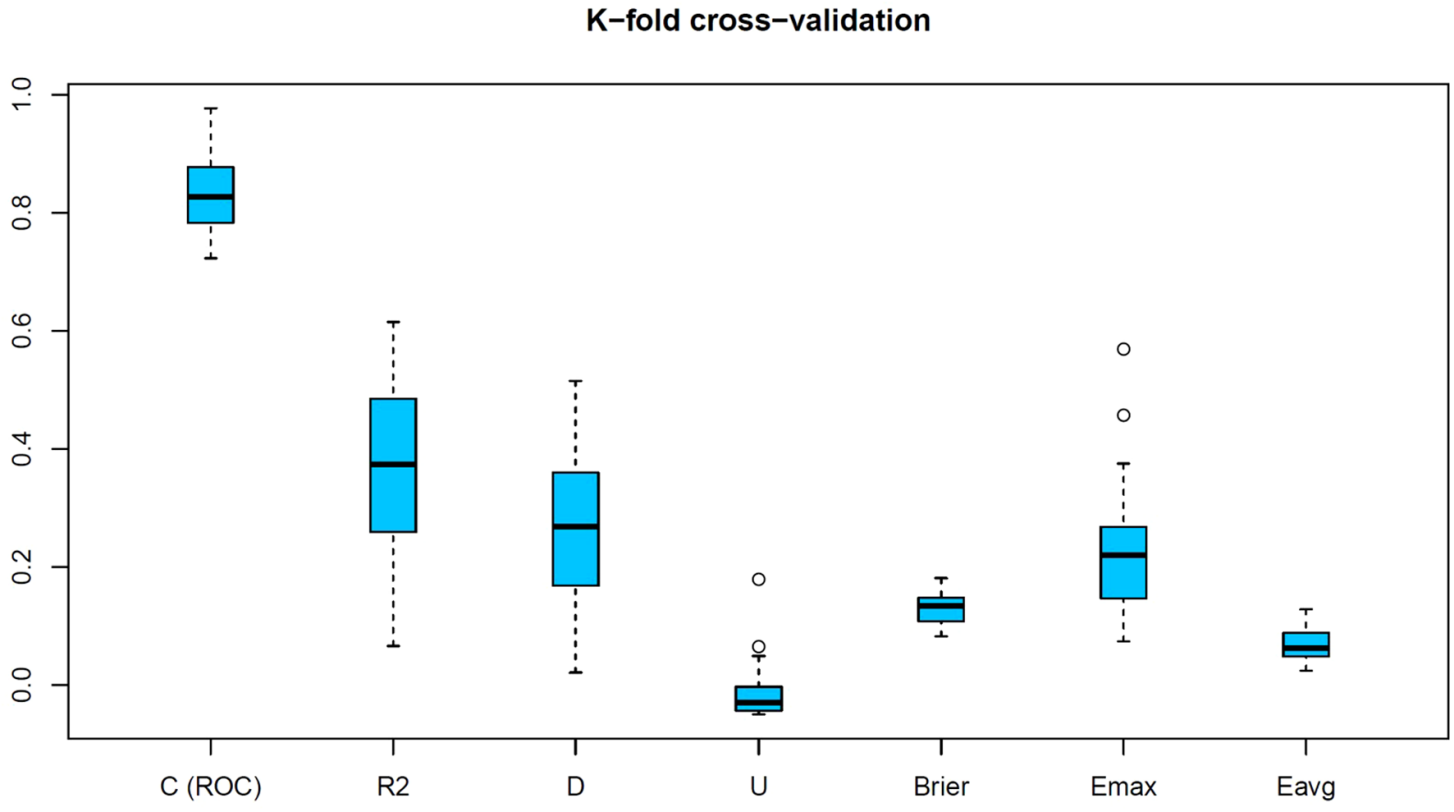
Ten-fold cross-validation results of the nomogram model. The boxplot shows the distribution of performance metrics across 10 validation folds. The median values (with ranges) were as follows: C-index, 0.80 (0.70- 0.92); Brier score, 0.10 (0.06- 0.14); Eavg, 0.02 (0.00- 0.06); Emax, 0.16 (0.16- 0.24); U, 0.14 (0.10- 0.18). The results demonstrate that the model exhibits good discriminative ability and acceptable calibration.

**Figure 4:**
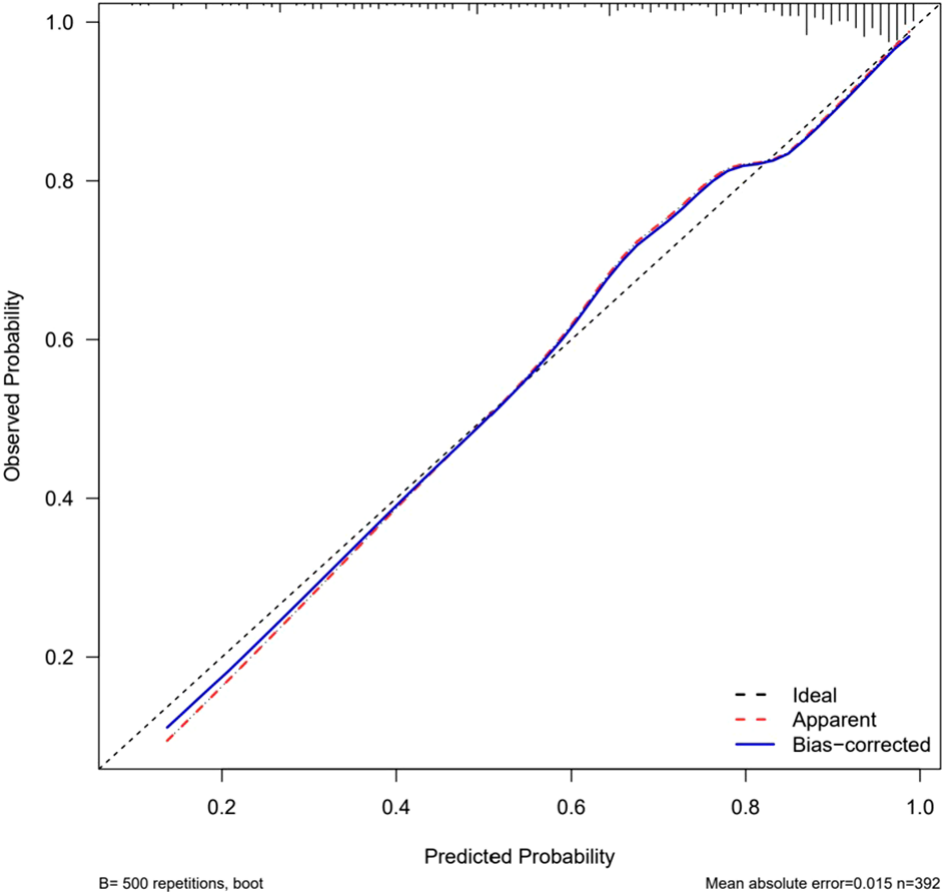
Bootstrap-corrected calibration curve of the nomogram model. The x-axis represents the predicted probability of peripheral AS, and the y-axis represents the observed frequency. The dashed diagonal line indicates ideal calibration. The bias-corrected curve (solid line) demonstrates acceptable agreement with the ideal line.

**Figure 5:**
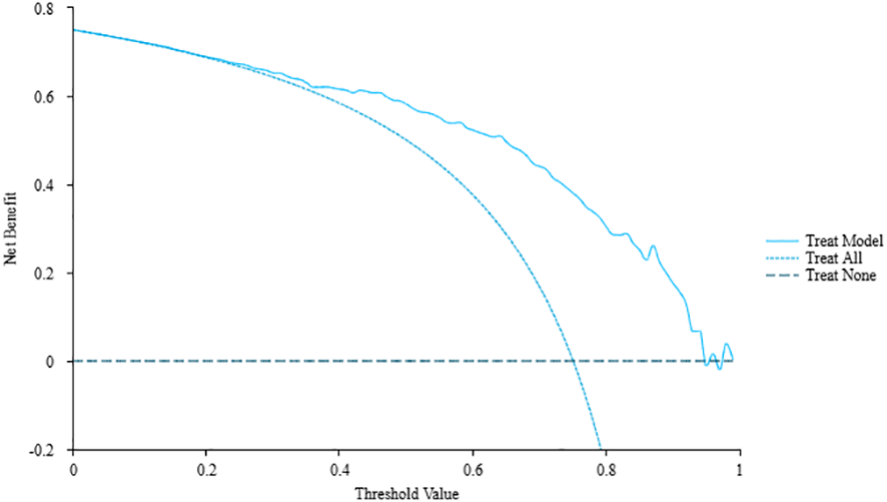
Decision curve analysis of prediction mode. The results showed that within a threshold range of 0-94%, the clinical net benefit of the model was consistently greater than 0, and the smaller the risk threshold, the greater the clinical net benefit, with a maximum value of 0.75.

Based on the independent risk factors identified by multivariate logistic regression analysis, we constructed three models incorporating Age to evaluate diagnostic efficacy. The AUC values for the three models, included Age only, FCP+ Age, and CVAI+ FCP+ Age, were 0.814 (95% CI: 0.765-0.863), 0.825 (95% CI: 0.776-0.874), and 0.837 (95% CI: 0.790-0.884), with a sensitivity of 87.4%, 85.0%, 87.1% and a specificity of 62.2%, 68.4%, 68.4%, respectively (**Figure 1**). Pairwise DeLong tests revealed that the AUC of the combined model (Age + FCP + CVAI, AUC = 0.837) was significantly larger than those of both the Age + FCP model (AUC difference = 0.0120, 95% CI: 0.000–0.024, p = 0.0431) and Age alone (AUC difference = 0.0229, 95% CI: 0.003–0.043, p = 0.0274). No significant difference was observed between Age alone and the Age + FCP model (p = 0.2295). A nomogram model was constructed based on the above three indicators to visualize the risk of AS in early-stage T2DM-DKD patients (**Figure 2**). With 10-fold cross-validation, the model demonstrated good overall discriminative ability, as indicated by a median C-index of 0.80 (range: 0.70- 0.92), a median Brier score of 0.10, a median estimated calibration error (Eavg) of 0.02, a median maximal calibration error (Emax) of 0.16, and a median calibration reliability index (U) of 0.14, indicating good overall discriminative ability of the model (**Figure 3**). The bootstrap-corrected calibration curve showed acceptable agreement between predicted probabilities and observed frequencies (**Figure 4**). DCA of the nomogram model indicated that within a threshold range of 0-94%, the clinical net benefit of the model was consistently greater than 0, and the smaller the risk threshold, the greater the clinical net benefit, with a maximum value of 0.75 (**Figure 5**).

## 4 Discussion

This is a single-center, retrospective clinical study based on data from hospitalized patients in China, focusing on early-stage T2DM-DKD Patients. We systematically explored the risk factors for peripheral AS and constructed an individualized prediction model incorporating CVAI, Age, and FCP for peripheral AS in early-stage T2DM-DKD Patients. The results showed that the detection rate of peripheral AS among the 392 early-stage T2DM-DKD patients was 75%, suggesting that vascular pathology is already widespread at the microalbuminuria stage, with a prominent phenomenon of cardio-renal comorbidity. This finding is highly consistent with the recent concept of the cardiovascular-kidney-metabolic (CKM) syndrome (16, 17). Proteinuria, as an early marker of DKD and an independent risk factor for CVD (18), should be regarded as an early warning sign of cardio-renal comorbidity. Multivariate logistic regression analysis revealed that elevated CVAI, increased Age, and decreased FCP were identified as independent risk factors for peripheral AS in patients with early-stage T2DM-DKD after adjusting for confounding factors. We constructed three models incorporating Age to evaluate diagnostic efficacy. The AUC values for the three models, included Age only, FCP+ Age, and CVAI+ FCP+ Age, were 0.814, 0.825, and 0.837, respectively. Delong test indicated that adding CVAI to the model containing Age and FCP provides a statistically significant but modest improvement in discriminative ability, whereas the addition of FCP alone does not significantly improve discrimination beyond Age alone.

Visceral adipose tissue is not merely an energy storage organ but also an active endocrine organ, and its dysfunction serves as a pivotal pathophysiological hub linking metabolic disorders, kidney damage, and AS (19, 20). Traditional indicators such as BMI and WC cannot accurately reflect the body fat distribution characteristics of Asian populations, while CT/MRI assessment of visceral fat has limitations including high cost and radiation exposure. CVAI integrates five indicators, namely Age, BMI, WC, TG, and HDL-C which specifically designed for Asian populations. It provides a stable, non-invasive, cost-effective, and reproducible assessment of visceral fat accumulation. In recent years, multiple large-scale cohort studies have confirmed that CVAI can effectively assess visceral obesity and predict the risk of DM and CVD (21–23). In the population with CKM syndrome, CVAI also serves as an effective surrogate marker of cardiometabolic risk (24, 25). In our study, we introduced CVAI into the early-stage DKD population, further expanding its application value in the context of cardio-renal comorbidity. The results showed that for each 1-SD increase in CVAI, the risk of peripheral AS increased by 3.4% (OR = 1.034, 95% CI: 1.010-1.058), independent of traditional risk factors such as age, duration of DM, and blood lipid levels. These findings further confirmed the applicability of CVAI in Asian populations with DKD and validating its value in evaluating metabolic cardiovascular disease risk. The underlying mechanisms may be as follows: Visceral adipose tissue releases free fatty acids, inflammatory cytokines, and adipokines, which induce chronic low-grade inflammation, oxidative stress, and IR, impair vascular endothelial function, and promote foam cell formation and plaque progression (26, 27). At the same time, it exacerbates renal ischemia, hypoxia, and mesangial proliferation, thereby driving the progression of DKD. These processes collectively form a cascade reaction of “visceral adiposity- metabolic disorder- cardiorenal injury” (28).

Age is a traditional risk factor for AS. Advancing age leads to decreased vascular elasticity, impaired endothelial repair capacity, and increased oxidative stress and inflammatory burden, thereby promoting arterial remodeling and plaque formation (29, 30). In the context of DKD, long-term glucolipid metabolic disorders and renal ischemia and hypoxia further accelerate vascular aging, making the predictive effect of age more prominent (31). Our study showed that each one-year increase in age was associated with a 5.4% increase in the risk of peripheral AS (OR = 1.054, 95% CI: 1.018-1.090). We acknowledge that while statistical multicollinearity (VIF < 5) was not severe, biological overlap between CVAI and Age exists, as Age is a component of the CVAI formula. Despite the biological interrelationship, multivariate logistic regression identified both CVAI and Age as statistically significant independent predictors of peripheral AS (CVAI: OR = 1.034, 95% CI: 1.010-1.058; Age: OR = 1.054, 95% CI: 1.018-1.090). This suggests that CVAI captures visceral adiposity-related risk information that is not fully accounted for by Age alone. This finding is consistent with the results of Shen et al. (24) based on the nationwide CHARLS cohort study (n = 7,744, follow-up of 9 years), and Liu et al. (32) based on a 10-year prospective cohort study (n = 6393). A striking finding of this study is that Age alone (AUC = 0.814) far outperforms CVAI alone (AUC = 0.567) in predicting peripheral AS, despite CVAI being a composite indicator that includes Age. Several explanations are plausible. First, Age is a non-modifiable, cumulative risk factor that captures lifelong exposure to vascular injury, including but not limited to metabolic insults. CVAI, in contrast, reflects a single time-point measurement of visceral adiposity, which is subject to short-term fluctuations and medication effects. AS is a slowly progressing process. Plaque burden is more closely related to long-term metabolic exposure than to a single measurement. As an inherent component of CVAI, Age may partially explain its stronger predictive ability. Second, the contribution of Age within CVAI is weighted for predicting visceral adiposity, not for directly predicting AS. Therefore, extracting Age from CVAI does not guarantee superior predictive power for AS. Third, the modest individual performance of CVAI suggests that visceral adiposity alone is a relatively weak standalone predictor in early DKD patients, possibly because other age-related mechanisms (e.g., vascular stiffening, impaired repair capacity) dominate. Importantly, the aim of our study was not to claim that CVAI and Age are biologically independent of each other, but rather to evaluate whether CVAI provides incremental predictive value for AS beyond the traditional risk factor of Age. The result of pairwise DeLong tests indicated adding CVAI to the model containing Age and FCP provides a statistically significant but modest improvement in discriminative ability, whereas the addition of FCP alone does not significantly improve discrimination beyond Age alone. Thus, CVAI should be interpreted as a synergistic contributor rather than a standalone powerful predictor in this specific population.

The present study demonstrated that a decreased FCP level was an independent protective factor against peripheral AS (OR = 0.771, 95% CI: 0.608-0.978), suggesting that insufficient endogenous insulin secretion is associated with an increased risk of peripheral AS. This finding warrants further investigation. FCP is a stable indicator of pancreatic β-cell function that is not interfered with by exogenous insulin. In patients with T2DM receiving exogenous insulin therapy, C-peptide levels can more accurately reflect endogenous insulin secretory capacity. Previous studies have suggested that C-peptide may exert vascular protective effects independently of insulin through mechanisms such as anti-inflammatory and anti-oxidative stress actions, rather than serving merely as a marker of insulin secretion. A decline in C-peptide level indicates pancreatic islet dysfunction, which may concurrently exacerbate metabolic disturbances, renal injury, and vascular pathology. However, some studies have proposed that C-peptide may contribute to the progression of DKD by promoting mesangial cell proliferation; therefore, the relationship between FCP and vascular complications may exhibit a U-shaped or J-shaped pattern (33, 34). The protective effect of FCP observed in this study may reflect the beneficial impact of relatively preserved pancreatic β-cell function on the vascular system in patients with early-stage DKD, although the underlying mechanisms require further validation through prospective studies. These findings provide novel insights into the protection of pancreatic islet function and the management of cardiovascular risk in patients with DKD.

In recent years, prediction models for diabetic complications have mostly focused on renal endpoints or major vascular events, and models covering both early-stage DKD and peripheral AS remain scarce. The present model fills this gap and provides a practical tool for integrated cardio-renal prevention and treatment. The advantages of this model are as follows: ①It incorporates CVAI, a novel indicator of body fat distribution, which is superior to traditional obesity indicators; ②It uses FCP to reflect pancreatic islet function, compensating for the limitations of using only blood glucose and HbA1c; ③The nomogram model constructed in this study includes three indicators, namely CVAI, Age, and FCP, which are readily available, easy to calculate, non-invasive, and cost-effective, making it suitable for routine use in both hospitalized and outpatient patients with early-stage DKD. This model enables individualized risk scoring, helping clinicians rapidly identify high-risk individuals and initiate early lifestyle interventions, as well as optimize glucose-lowering, blood pressure-lowering, lipid-lowering, and kidney-protective regimens. This is particularly relevant to China’s national conditions, characterized by a large population of patients with early-stage DKD and limited screening resources, and has practical significance for reducing the CVD burden in Chinese patients with DKD.

This study has the following limitations: First, this was a single-center retrospective study, which may have introduced selection bias and information bias. We have noted that the AS+DKD group exhibited lower TC, TG, LDL, and BMI, but higher HDL, which appears counterintuitive. This pattern is most likely explained by reverse causality and the higher rate of statin use in the AS+DKD group (35.0% vs. 9.2%). Although we found that CVAI, Age, and FCP remained independently associated with peripheral AS after adjusting for statin use as a covariate, residual confounding due to indication and medication adherence cannot be completely excluded, thus extrapolation to community populations should be made with caution. Second, the sample size was limited, and the model requires external validation in multicenter, large-sample, prospective cohort studies. Third, biological markers such as inflammatory factors, oxidative stress, and adipokines were not measured, resulting in insufficient exploration of the molecular mechanisms by which CVAI mediates AS. Finally, long-term follow-up was not conducted, making it impossible to determine the predictive value of CVAI for hard cardiovascular endpoints. Future research should include multicenter prospective studies to validate the model’s performance, further explore the pathways through which visceral adipokines contribute to DKD complicated by peripheral AS, and conduct intervention studies targeting CVAI to evaluate whether weight loss and metabolic optimization reduce the risk of vascular events.

## 5 Conclusion

In conclusion, elevated CVAI, increased Age, and decreased FCP were independent risk factors for peripheral AS in early-stage T2DM-DKD patients. The nomogram model constructed based on CVAI, Age, and FCP demonstrates good predictive performance and strong clinical operability, and can be used for cardiovascular risk stratification and individualized early warning in patients with early-stage DKD. Integrating CVAI into routine clinical evaluation, emphasizing precise management of visceral adiposity, preserving pancreatic β-cell function, and implementing early intervention for vascular lesions may help interrupt the vicious cycle between DKD and AS, reduce the risk of cardiovascular, and improve long-term patient outcomes. It is worth noting that, while CVAI alone is a weak predictor of peripheral AS in early T2DM-DKD, it contributes meaningfully to risk stratification when combined with Age and FCP. The proposed nomogram may facilitate early identification of high-risk patients, but the individual predictive limitations of CVAI should not be overlooked.

## Data Availability

The raw data supporting the conclusions of this article will be made available by the authors, without undue reservation.
